# HER2-low-positive breast cancer: evolution from primary tumor to residual disease after neoadjuvant treatment

**DOI:** 10.1038/s41523-022-00434-w

**Published:** 2022-05-20

**Authors:** Federica Miglietta, Gaia Griguolo, Michele Bottosso, Tommaso Giarratano, Marcello Lo Mele, Matteo Fassan, Matilde Cacciatore, Elisa Genovesi, Debora De Bartolo, Grazia Vernaci, Ottavia Amato, Francesca Porra, PierFranco Conte, Valentina Guarneri, Maria Vittoria Dieci

**Affiliations:** 1 Department of Surgery, Oncology and Gastroenterology (DISCOG), University of Padova, 35128 Padova, Italy; 2 Medical Oncology 2, Istituto Oncologico Veneto IOV-IRCCS, 35128 Padova, Italy; 3 Surgical Pathology Unit, University Hospital of Padua, 35121 Padua, Italy; 4 Department of Medicine (DIMED), Surgical Pathology & Cytopathology Unit, University of Padua, 35121 Padua, Italy; 5 Istituto Oncologico Veneto IOV-IRCCS, Padua, Italy; 6 Department of Pathology and Molecular Genetics, Treviso General Hospital, Treviso, Italy

**Keywords:** Diagnostic markers, Breast cancer

## Abstract

Approximately a half of breast tumors classified as HER2-negative exhibit HER2-low-positive expression. We recently described a high instability of HER2-low-positive expression from primary breast cancer (BC) to relapse. Previous studies reporting discordance in HER2 status between baseline biopsy and residual disease (RD) in patients undergoing neoadjuvant treatment did not include the HER2-low-positive category. The aim of this study is to track the evolution of HER2-low-positive expression from primary BC to RD after neoadjuvant treatment. Patients undergoing neoadjuvant treatment with available baseline tumor tissue and matched samples of RD (in case of no pCR) were included. HER2-negative cases were sub-classified as HER2-0 or HER2-low-positive (IHC 1+ or 2+ and ISH negative). Four-hundred forty-six patients were included. Primary BC phenotype was: HR-positive/HER2-negative 23.5%, triple-negative (TN) 35%, HER2-positive 41.5%. HER2-low-positive cases were 55.6% of the HER2-negative cohort and were significantly enriched in the HR-positive/HER2-negative vs. TN subgroup (68.6% vs. 46.8%, *p* = 0.001 *χ*^2^ test). In all, 35.3% of non-pCR patients (*n* = 291) had a HER2-low-positive expression on RD. The overall rate of HER2 expression discordance was 26.4%, mostly driven by HER2-negative cases converting either from (14.8%) or to (8.9%) HER2-low-positive phenotype. Among HR-positive/HER2-negative patients with HER2-low-positive expression on RD, 32.0% and 57.1% had an estimated high risk of relapse according to the residual proliferative cancer burden and CPS-EG score, respectively. In conclusion, HER2-low-positive expression showed high instability from primary BC to RD after neoadjuvant treatment. HER2-low-positive expression on RD may guide personalized adjuvant treatment for high-risk patients in the context of clinical trials with novel anti-HER2 antibody-drug conjugates.

## Introduction

Breast cancer is recognized as a highly heterogeneous disease in terms of biological features, prognosis and treatment sensitivity. In the past decades access to anti-HER2 drugs has been driven by the dichotomy between HER2-positive and HER2-negative breast cancer established in the context of pivotal trials of trastuzumab, however recent findings have challenged this dogma. In particular, early-phase clinical trials reported promising anti-tumor activity of the anti-HER2 antibody-drug conjugates (ADC) Trastuzumab-Deruxtecan and Trastuzumab-Duocarmazine in patients traditionally classified as having HER2-negative breast cancer though exhibiting HER2-low-positive expression (IHC scores 1+ or 2+ in the absence of *HER2* gene amplification by ISH)^1,2^. In addition, recently, a phase II study treating 48 heavily pre-treated hormone-receptor (HR)-positive-HER2-low-positive advanced breast cancer patients with the anti-HER2/HER3 ADC zenocutuzumab (in combination with the last endocrine agent on which the patients had previously progressed immediately before the study entry), reported promising preliminary results in terms of clinical activity and safety^3^. These findings provided the proof of principle that novel anti-HER2 ADCs may exploit, to be active, the mere presence of the target, rather than a proven oncogene addiction to HER2. Two phase III clinical trials randomizing HER2-low-positive metastatic breast cancer patients to receive either Trastuzumab-Deruxtecan or treatment of physician’s choice are currently ongoing and results are pending (NCT03734029—Destiny-Breast04 met its primary endpoint—data not presented yet, NCT04494425—Destiny-Breast06). In addition, several other diverse combinations of anti-HER2 agents plus endocrine therapy/CDK 4/6 inhibitors/immunotherapy/other targeted agents are currently being tested in early-phase clinical trials, thus emphasizing the fervent interest on this dawning breast cancer subtype^4^. On the same grounds, ongoing efforts have been directed towards uncovering whether HER2-low-positive tumors may retain possible unique traits^5–7^, however, so far, solid evidence supporting HER2-low-positive breast cancer as a distinct biological and/or clinical entity is lacking.

We recently reported a high instability of HER2-low-positive expression from primary breast cancer to relapse^8^, suggesting that a not negligible proportion of patients originally classified as HER2-0 becomes HER2-low-positive at recurrence thus potentially expanding their therapeutic options.

Neoadjuvant treatment currently represents a widely adopted strategy for patients with early breast cancer given the well-acknowledged benefits in terms of expansion of locoregional treatment options, in-vivo evaluation of treatment sensitivity and the possibility to tailor post-neoadjuvant approach based on the pathological response after neoadjuvant treatment^9,10^. In this particular context, FDA has recently endorsed to select high-risk patients to be enrolled in adjuvant escalation trials based on the presence of residual disease after neoadjuvant therapy^11^. Interestingly, in patients undergoing neoadjuvant treatment and failing to achieve a pCR, the finding of discordance in HR and HER2 status from baseline biopsy to residual disease has been reported as a relatively frequent phenomenon^12–16^. However, previous studies did not include the emerging HER2-low-positive category in their evaluation.

In the present work, we investigated the evolution of HER2 expression from primary breast cancer to matched samples of residual disease in a large cohort of breast cancer patients undergoing neoadjuvant chemotherapy.

## Results

### Patient cohorts and clinicopathologic features

**Fig. 1:**
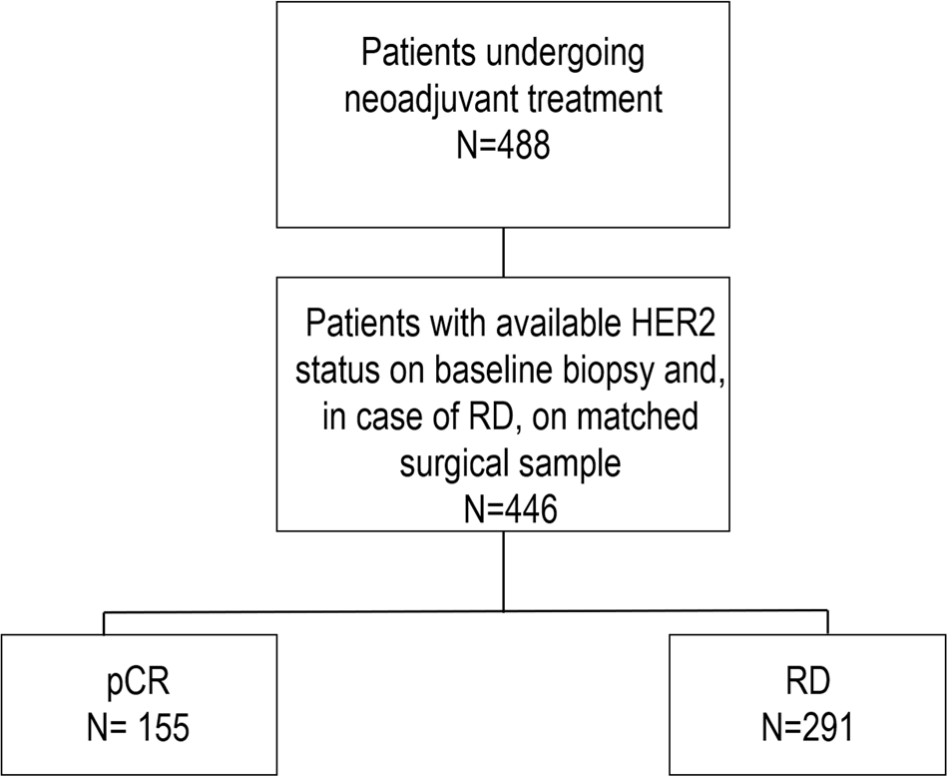
Flow diagram of the study. This diagram shows the study scheme. *N* number, pCR pathologic complete response, RD residual disease.

A total of 446 breast cancer patients undergoing neoadjuvant chemotherapy were included, as shown in Fig. 1. Table 1 shows main clinicopathologic features of the overall cohort. The majority of patients had tumors of ductal histology (*n* = 397, 89.0%) and poor differentiation (G3, *n* = 326, 70.9%) on baseline biopsy and almost 94% of patients had clinical stage II–III breast cancer (stage II: *n* = 259, 58,1%; stage III: *n* = 159, 35.7%). Tumor phenotypes on baseline biopsy were distributed as follows: HR-positive/HER2-negative 23.5% (*n* = 105), triple-negative 35% (*n* = 156) and HER2-positive 41.5% (*n* = 185; HER2-positive/HR-positive, *n* = 104; HER2-positive/HR-negative, *n* = 81). In the great majority of cases patients underwent anthracycline-taxane-based neoadjuvant chemotherapy (*n* = 354, 79.4%) and among HER2-positive patients, 86.5% received anti-HER2 blockade associated with neoadjuvant chemotherapy (*n* = 160). One-hundred fifty-five patients achieved pCR after neoadjuvant chemotherapy (34.8%). The distribution of pCR rates according to tumor phenotype is shown in Table 2. As expected, significantly higher rates of pCR were observed in triple-negative and HER2-positive subgroup as compared to HR-positive/HER2-negative subtype (38.5%, 45.9%, and 9.5% respectively, *p* < 0.001 *χ*^2^ test). Among 291 patients with residual disease after neoadjuvant therapy, the distribution of breast cancer phenotype was: HR-positive/HER2-negative 35.7% (*n* = 104), triple-negative 32% (*n* = 93) and HER2-positive 32.3% (*n* = 94; HER2-positive/HR-positive, *n* = 66; HER2-positive/HR-negative, *n* = 28). 17.9% of patients received further chemotherapy in the adjuvant setting (*n* = 80); among HER2-positive breast cancer patients, more than 90% received adjuvant anti-HER2 therapy (*n* = 177); 227 patients received adjuvant hormonal therapy.

**Table 1:** Main clinicopathologic characteristics.

	*N*	%	Median	(Q1–Q3)
Age			50.2	42.7–60.2
Hystology				
Ductal	397	89.0		
Lobular	28	6.3		
Other/NA	21	4.7		
Grading				
1	4	0.9		
2	89	20.0		
3	316	70.9		
NA	37	8.2		
Clinical TNM				
I	21	4.7		
II	259	58.1		
III	159	35.7		
NA	7	1.5		
Primary BC phenotype				
HR+/HER2−	105	23.5		
TN	156	35.0		
HER2+	185	41.5		
Neoadj. CT				
Anthra-Tax	354	79.4		
Tax	68	15.2		
Anthra	9	2.0		
Other/NA	15	3.4		
Neoadj. anti-HER2				
Trastuzumab	160	35.9		
Pathologic response				
pCR	155	34.8		
RD	291	65.2		

*Neoadj* neoadjuvant, *CT* chemotherapy, *anthra* anthracycline, *tax* taxane, *pCR* pathologic complete response, *RD* residual disease.

**Table 2:** Distribution of pCR rates according to tumor phenotype.

	pCR, *n* (%)	*p*-value
Primary BC phenotype		<0.001^a^
HR+ /HER2− (105)	10 (9.5)	
TN (156)	60 (38.5)	
HER2+ (185)	85 (45.9)	

*BC*, breast cancer, *HR*+ hormone-receptor positive, *HER2*– HER2-negative, *TN* triple-negative, *HER2*+ HER2-positive, *pCR* pathologic complete response.

^a^*χ*^2^ test.

### Features of HER2-low-positive breast cancer in baseline biopsy and residual disease

Among HER2-negative cases (*n* = 261), the distribution of breast cancer phenotype on baseline biopsy according to HER2 expression was as follows: HER2-0 44.4% (*n* = 116), HER2-low-positive 55.6% (*n* = 145). A higher proportion of HER2-low-positive cases was observed in HR-positive/HER2-negative subgroup as compared to triple-negative subtype (68.6% vs. 46.8%, respectively, *p* = 0.001 *χ*^2^ test), as shown in Table 3.

**Table 3:** Distribution of primary breast cancer phenotype according to HER2 expression.

	HER2–0, *n* (%)	HER2-LOW-POSITIVE, *n* (%)	*p-*value
Primary BC phenotype			0.001^a^
HR+/HER2–	33 (31.4)	72 (68.6)	
TN	83 (53.2)	73 (46.8)	

*BC* breast cancer, *HR*+ hormone-receptor positive, *HER2*– HER2-negative, *TN* triple-negative.

^a^*χ*^2^ test.

In the subgroup of patients failing to achieve pCR (*n* = 291), the proportion of HER2-low-positive cases on residual disease was 35.3% (*n* = 103), corresponding to 52.3% of the HER2-negative cohort. Similar to the basal tissue samples, a highly significant association between HER2-low-positive expression on residual disease and HR status was observed (HER2-low-positive proportion among HR-positive and triple-negative cases: 65.4% vs. 37.6%, respectively, *p* < 0.001 *χ*^2^ test).

HER2 expression distribution according to tumor phenotype on RD in the HER2-negative cohort is shown in Table 3.

**Fig. 2:**
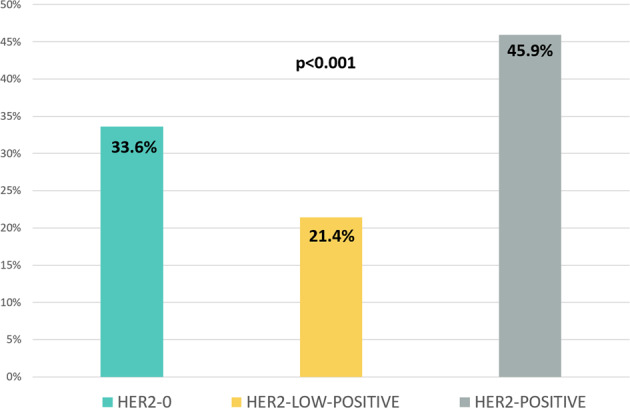
pCR rates according to HER2 expression. This bar chart shows pCR rates across subgroups defined by HER2 expression (*p*-value obtained with *χ*^2^ test).

A significant association was observed between HER2 expression and the probability to achieve pCR. In particular, HER2-low-positive breast cancer was associated with the lowest rate of pCR, followed by HER2-0 and HER2-positive tumors (pCR rates HER2-low-positive vs. HER2-0 vs. HER2-positive: 21.4% vs. 33.6% vs. 45.9%, respectively, *p* < 0.001 *χ*^2^ test), as shown in Fig. 2. In addition, when focusing on HER2-negative cases, the significance of this relationship was maintained (HER2-0 vs. HER2-low-positive, *p* = 0.035 *χ*^2^ test). However, when considering each phenotypic subset (HR-positive/HER2-negative and TN), the association between lower pCR rates and HER2-low-positive phenotype was no longer significant (pCR rates in HR-positive/HER2-0 vs. HR-positive/HER2-low-positive: 12.1% vs. 8.3%, *p* = 0.721 *χ*^2^ test; TN/HER2-0 vs. TN/HER2-low-positive: 42.2% vs. 34.2%, *p* = 0.327 *χ*^2^ test).

In order to evaluate the proportion of HR-positive/HER2-low-positive breast cancer patients with high-risk features besides the failure to achieve pCR, the validated RPCB and CPS-EG scores were computed. Among patients with HR-positive/HER2-negative breast cancer and residual disease, RPCB and CPS-EG scores were available for 70 and 84 cases, respectively. The distribution of RPCB and CPS-EG classes according to HER2 expression on residual disease in shown in Table 4. In particular, among HR-positive/HER2-low-positive patients failing to achieve pCR with data available, 32.0% and 57.1% of had an estimated high risk of relapse based on RPCB class 3 and CPS-EG score ≥3, respectively.

**Table 4:** Distribution of breast cancer phenotype on residual disease according to HER2 expression.

	HER2–0, *n* (%)	HER2-low-positive, *n* (%)	*p*-value
RD phenotype			<0.001^a^
HR+/HER2–	36 (34.6)	68 (65.4)	
TN	58 (62.4)	35 (37.6)	

*RD* residual disease, *HR+* hormone-receptor positive, *HER2*– HER2-negative, TN triple-negative.

^a^*χ*^2^ test.

### HER2 evolution from baseline biopsy to residual disease after neoadjuvant treatment

**Fig. 3:**
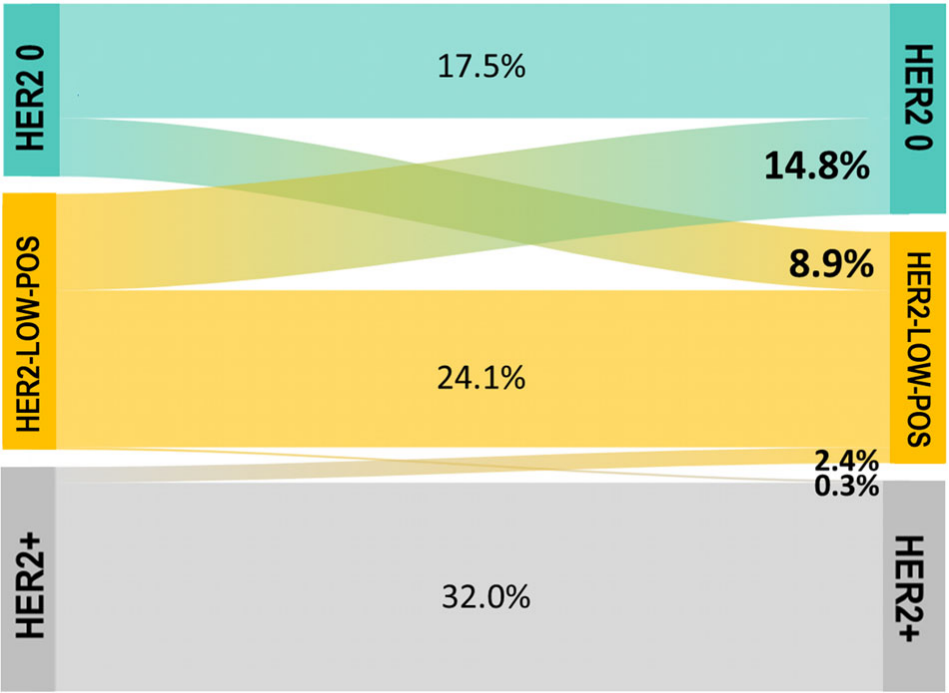
Evolution of HER2 expression. This Sankey diagram shows the evolution of HER2 expression from baseline biopsy to residual disease after neoadjuvant chemotherapy in patients failing to achieve pCR.

The evolution of HER2 expression from baseline biopsy to residual disease after neoadjuvant chemotherapy in patients failing to achieve pCR is shown in Fig. 3. The overall rate of HER2 discordance was 26.4%, mostly represented by cases switching from HER2-0 to HER2-low-positive (*n* = 26, 8.9%) and from HER2-low-positive to HER2-0 phenotype (*n* = 43, 14.7%). In detail, among patients with HER2-0 phenotype on baseline biopsy, 33.8% (*n* = 26) experienced a conversion to HER2-low-positive phenotype, while 37.7% (*n* = 43) of HER2-low-positive breast cancer patients showed a conversion in the opposite direction. HER2-positive status was the most stable, with 2.7% (*n* = 8) of patients exhibiting either a gain (*n* = 1) or loss (*n* = 7) of HER2 positivity.

**Fig. 4:**
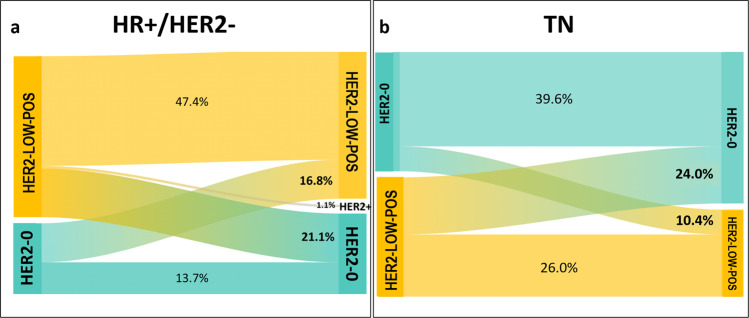
Evolution of HER2 expression according to breast cancer subtype. These Sankey diagramas show the evolution of HER2 expression from baseline biopsy to residual disease after neoadjuvant chemotherapy in patients failing to achieve pCR in the HER2-negative cohort according to breast cancer phenotype. **a** Evolution of HER2 expression in HR+/HER2+subtype; **b** Evolution of HER2 expression in TN subtype. HR+ hormone-receptor positive, TN triple-negative.

The evolution of HER2 expression in the HER2-negative cohort according to breast cancer phenotype is shown in Fig. 4. In particular, among HR-positive/HER2-negative breast cancer patients failing to achieve pCR (*n* = 95), the overall rate of HER2 discordance was 39%. In detail, 16.8% (*n* = 16) patients showed a conversion from HER2-0 primary breast cancer to HER2-low-positive residual disease, 21.1% showed a switch in the opposite direction (*n* = 20) and 1.1% (*n* = 1) exhibited the acquisition of HER2-positive status. Among triple-negative breast cancer patients failing to achieve pCR (*n* = 96), 10.4% (*n* = 10) showed a conversion from HER2-0 primary breast cancer to HER2-low-positive residual disease and 24.0% (*n* = 23) showed a switch in the opposite direction. None of the triple-negative breast cancer patients exhibited HER2-positivity gain.

Additionally, we evaluated the switch of hormone receptors from baseline biopsy to matched RD samples. The total rate of hormone-receptor discordance was 8.9% (*n* = 26). In particular, ER discordance rate was 6.2% (*n* = 18), with 2.0% of ER loss (*n* = 6) and 4.2% (*n* = 12) of ER gain; PgR discordance rate was 20.2% (*n* = 58), encompassing 12.9% rate of PgR loss (*n* = 37) and 7.3% of PgR gain (*n* = 21).

### Exploratory survival analysis

Exploratory survival analysis did not reveal any statistically significant disease-free-survival (DFS) difference according to baseline HER2 expression. In particular, similar DFS rates were observed between HER2-0 and HER2-low-positive BC cohorts (HR 0.79, 95% CI 0.51–1.21, *p* = 0.27 log-rank test and Cox-regression model; Supplementary Fig. 1A). Similarly, no differences in DFS were observed according to HER2 expression when separately evaluating HR-positive/HER2-negative subgroup (HR 0.74, 95% CI 0.39–1.41, *p* = 0.35 log-rank test and Cox-regression model, Supplementary Fig. 1B) and triple-negative subgroup (HR 0.92, 95% CI 0.52–1.65, *p* = 0.79 log-rank test and Cox-regression model; Supplementary Fig. 1C).

In addition, when evaluating the potential prognostic impact of HER2 expression change from baseline biopsy to residual disease, focusing on HER2-negative cases, there was no DFS difference for concordance vs. discordance (HR 1.26, 95% CI 0.81–1.95, *p* = 0.29 log-rank test and Cox-regression model), concordant HER2–0 vs. gain of HER2-low-positive expression (HR 1.10, 95% CI 0.57–2.12, *p* = 0.77 log-rank test and Cox-regression model) or concordant HER2-low-positive vs. loss of HER2-low-positive expression (HR 1.43, 95% CI 0.79–2.57, *p* = 0.23 log-rank test and Cox-regression model), as shown in Supplementary Fig. 2A–C, respectively.

## Discussion

In our large cohort of 446 breast cancer patients undergoing neoadjuvant treatment, we confirmed the strong relationship between HER2-low-positive breast cancer and HR-positive status, thus strengthening the possible crucial role or ER signaling in shaping HER2-low-positive breast cancer biology^5,17–21^. These findings are mirrored in recently reported gene expression analyses, that revealed HR-positive/HER2-negative cases with HER2-low-positive expression being more likely profiled as Luminal by PAM50-based intrinsic subtyping^5,6,22^.

In addition, we observed significantly lower pCR rates in patients with HER2-low-positive phenotype as compared to HER-0. This observation appears consistent with what has been recently reported in a pooled-analysis of individual data from four prospective trials, where HER2-low-positive patients experienced significantly lower pCR than those with HER2-0 breast cancer in the overall cohort and in HR-positive subgroup^6^. However, in our study, when assessing pCR rates separately in HR-positive/HER2-negative and TN subgroups, the association between HER2 expression and pCR was no longer significant, thus suggesting that, in our HER2-negative cohort, the major determinant of chemo-sensitivity was HR status rather than HER2 expression. Indeed, as expected, we observed HR-positive/HER2-negative patients having significantly lower pCR than the TN subgroup and, given the significant enrichment of the HER2-low-positive subgroup for HR-positive cases, these latter could have driven the lower rate of pCR of the overall HER2-low-positive cohort.

The main objective of this work was to explore the evolution of HER2-low-positive expression from baseline tumor to residual disease in patients undergoing neoadjuvant chemotherapy, by adopting a HER2-based three-tier algorithm. We observed a 26.4% overall rate of HER2 discordance from baseline biopsies to residual disease samples and this phenomenon mostly reflected the conversion to or from HER2-low-positive expression. Of note, we previously reported HER2 expression being highly unstable during disease evolution from primary breast cancer to relapse, mainly due to HER2-low-positive cases switching either from or to HER2-0 expression and findings from the present work solidify the great instability of HER2-low-positive expression in a different setting. Interestingly, all HER2-positive breast cancer patients exhibiting HER2-loss after neoadjuvant treatment converted to HER2-low-positive phenotype on residual disease, while none of them experienced a complete loss of HER2 expression.

There are several implications of our results.

Firstly, this susceptibility of HER2-low-positive expression to conversion after the exposure to neoadjuvant treatment adds to available evidence suggesting HR and/or HER2 status discordance from primary tumor to residual disease after neoadjuvant treatment as a relatively frequent phenomenon^12–16^. In this context, if, from one hand, our findings emphasize the importance to re-profile the tumor on residual disease, on the other, they support the inclusion of the HER2-low-positive category in this evaluation. It should however be noted that in our cohort, all patients underwent chemotherapy, thus precluding the possibility to uncovering whether the instability of HER2-low-positive expression reflects a genuine shift as a consequence of chemotherapy exposure or rather an analytical distortion. In this context, although the diagnostic accuracy of HER2 evaluation on core-needle biopsies has been suggested to be high as compared to surgical specimen^23–25^, this conclusion has been drawn by focusing on the dichotomization between HER2-positive vs. HER2-negative status. We have previously reported higher proportion of HER2-low-positive cases when primary tumor phenotype was assessed on core-needle biopsies as compared to treatment-naive surgical specimens, with pre-analytical variables and intratumor heterogeneity of HER2 expression both representing possible contributing factors to such analytical variability. Of course, this area might warrant further investigation^8^.

Secondly, if ongoing late-phase trials will confirm the positive results from early-phase clinical trials of novel anti-HER2 strategies for HER2-low-positive advanced breast cancer patients (NCT03734029 —Destiny-Breast04 met its primary endpoint—data not presented yet, NCT04494425—Destiny-Breast06), it is expected a rapid transfer of this experimental scenario in the early setting. In this context, our findings anticipate the forthcoming and, at that point, imperative need to broaden the pool of patients who may get access to anti-HER2 blockade in the context of clinical trials as well as proper selecting those who may potentially derive the greatest benefit from these novel strategies^26^. Indeed, we identified more than one third of patients with HER2-0 phenotype at baseline showing a conversion to HER2-low-positive expression after neoadjuvant treatment, thus suggesting that the evaluation of HER2 expression on residual disease may allow the access to potentially effective novel treatment strategies in a not negligible proportion of patients who would otherwise be excluded based on the primary tumor phenotype. In addition to that, when focusing on HR-positive/HER2- breast cancer patients showing HER2-low-positive expression on residual disease, we observed a high proportion of patients being classified as having high-risk features based on the previously validated prognostic scores RPCB and CPS-EG score. Indeed, while the mere presence of residual disease after neoadjuvant chemotherapy has been endorsed as a fit criterion for selecting high-risk triple-negative breast cancer patients for clinical trials testing escalated post-neoadjuvant strategies, this might not be the more reliable strategy for selecting high-risk patients with HR-positive/HER2- breast cancer, given the known sub-optimality of pCR as a surrogate prognostic biomarker in this breast cancer subtype^27^. Indeed, in recent years, several biomarkers considering not only the burden of residual disease but also its biology have been suggested as superior to pCR in terms of prognostic stratification. In this context, we decided to adopt CPS-EG score and RPCB for this purpose since they both have been validated specifically in the HR-positive/HER2- breast cancer subtype^17–20^, allowing us to identify HR-positive/HER2-low-positive breast cancer patients who may be defined at high risk and hence be potentially selected for post-neoadjuvant trials testing novel anti-HER2 ADCs as escalated strategies.

Another point deserving further discussion is that 7% of patients with HER2-positive breast cancer at baseline with no pCR, lost HER2 positivity, exhibiting HER2-low-positive phenotype on residual disease. Current evidence suggests that HER2-loss after neoadjuvant treatment may confer an additional negative prognostic trait in HER2-positive breast cancer patients already defined at high-risk of relapse based on the failure to achieve pCR^12,16^. Indeed, although subgroup analyses from the KATHERINE trial, which established TDM1 as the new standard of care in the post-neoadjuvant setting in patients failing to achieve pCR^28^, revealed that patients with HER2-loss at surgery still seemed to benefit from TDM1 over trastuzumab^29^, it is currently largely unknown whether those maintaining some level of HER2 expression (HER2-low-positive subgroup) may derive greater advantage by the administration of novel anti-HER2 ADCs given post-neoadjuvantly with respect to TDM1. Of course, translational analyses from an ongoing clinical trial comparing post-neoadjuvant TDM1 vs. Trastuzumab-Deruxtecan (NCT04622319) may be able to shed light in this unexplored area.

Exploratorily, we also performed an evaluation of hormone-receptor changes from baseline biopsy to residual disease after neoadjuvant therapy, observing an overall rate of discordance of 8.9%, thus further consolidating the value of biomarker status re-evaluation after neoadjuvant treatment in case of no-pCR. Within this framework, consistently with available evidence, we confirmed PgR being more prone to discordance than ER^24,30^.

Finally, an exploratory survival analysis was also conducted, which did not reveal any significant DFS difference between HER2-0 vs. HER2-low-positive expression at baseline, thus fueling the already existing uncertainty regarding the actual clinical distinctiveness of HER2-low-positive expression from a prognostic point of view^5–7,26,31^. Interestingly, HER2 expression discordance (nor either HER2-low-positive expression loss or gain) from baseline biopsy to residual disease after neoadjuvant chemotherapy did not retain any prognostic role in terms of DFS in HER2-negative breast cancer patients. These data stress the notion that the most relevant implication of retesting HER2 expression on residual disease by also including the HER2-low-positive category, might be that of enhancing the access to potentially effective drugs in patients at high-risk of relapse.

The present works presents several strengths. Firstly, this represents a study evaluating, in a large cohort of patients undergoing neoadjuvant chemotherapy, HER2 expression evolution from primary breast cancer to residual disease by incorporating the up-and-coming category of HER2-low-positive breast cancer. Secondly, although HER2 expression data reflected the local evaluation according to ASCO/CAP recommendations endorsed at the time of diagnosed, all cases diagnosed between 2007 and 2013 were reviewed to comply with the currently accepted 10% cutoff of cell staining for HER2 positivity, given that in 2007 this cutoff was raised to 30% before being restored to the original 10% in 2013. Thirdly, we decided to embrace the terminology recently suggested as a basis for a future international consensus for breast cancer classification according to HER2 expression^6^. Another possible strength of the present study is represented by the adoption of a 10% cutoff for distinguishing HR-positive vs. HR- cases. Indeed, although a major debate is still ongoing with regard to the optimal ER expression threshold for defining the access to endocrine therapy^32^ a mounting body of evidence suggests that patients harboring ER levels ranging from 1 to 10% (the so-called ER-low breast cancer) are phenotypically more similar to TN than HR-positive breast cancer^33–36^.

A final consideration regards the analytical reliability of IHC/ISH methods for detecting low levels of HER2 expression. In fact, on one hand, with the emergence of novel treatment strategies directed to patients with HER2-low-positive breast cancer, a stricter adherence to FDA/ASCO-CAP rules for HER2 scoring would be advisable, especially in the light of the suboptimal inter-pathologist agreement rates recently reported with regards to the distinction between IHC scores 0 and 1+^5^. On the other hand, the actual sensitivity of a semiquantitative assay, as IHC, in detecting low levels of HER2 expression may be questioned, and it is not inconceivable to hypothesize that a proportion of score = 0 by IHC may reflect an artefactual limitation rather than a genuine total absence of HER2 expression. On this ground, one might question to which extent low levels of HER2 expression should be considered too low to grant access to novel anti-HER2 agents. In this context, it might be worth investigating alternative techniques for the quantitative evaluation of HER2 expression, including those based on gene expression and proteomics analyses, some of which have already proven to be promising at this purpose^37–42^.

The major limitation of the present work is its retrospective nature, which may have been responsible for both selection and information bias. Another limitation is represented by the heterogeneity of neoadjuvant treatments. However, the impact of this flaw is downsized by the fact that almost 80% of the patients received anthracycline-taxane-based neoadjuvant chemotherapy, which currently represents the standard chemotherapy backbone in this setting. Finally, a central HER2 expression revision of all cases was not planned, however a good agreement with the original report was obtained after 100 random samples were reviewed by an expert pathologist in a blinded fashion.

In conclusion, we reported a remarkable instability of HER2 expression from primary breast cancer to residual disease in a large cohort of patients undergoing neoadjuvant chemotherapy, with HER2-low-positive expression instability being the major driver of such phenomenon. Our results encourage the reprofiling of residual disease by including the HER2-low category.

## Materials and methods

### Population

Breast cancer patients undergoing neoadjuvant chemotherapy at The Oncology Department of the Istituto Oncologico Veneto—IRCCS, Padova Italy, between January 2002 and June 2018 were identified. Cases for which HER2 status was available on baseline biopsy and, in case of residual disease, on matched surgical samples were included. Patients’ clinicopathologic features including age, stage at diagnosis, baseline HR status/expression and HER2 status and expression, type of neoadjuvant treatment, pathologic response (pCR vs. no-pCR), HR status/expression and HER2 status on residual disease (in case of no-pCR) were recorded.

### Pathology

Cases were considered as HER2-positive in case of IHC score 3+ and/or *HER2* gene amplification by ISH, HER2-low-positive in case of IHC scores 1+ or 2+ in the absence of gene amplification by ISH and HER2-0 in case of IHC score zero. In the present study we complied with the terminology proposed by Denkert et al. in their pivotal study on HER2-low-positive landscape in early breast cancer^6^. HER2 expression was retrospectively retrieved from pathology records and evaluated according to ASCO/CAP recommendations in place at the time of diagnosis (the IHC assays for HER2 adopted were all FDA approved and were: until 2008 Hercept-Test®, DAKO [ref. number 2007333; 1:1000 dilution]; 2009–2011 Pathway®, Ventana [clone 4B5] [ref. number 780–4422; 1:3000 dilution]; since 2012 Bond Oracle®, Leica [clone CB11] [ref. number 780–4422, 1:1000 dilution])). However, given the temporary endorsement of the 30% cutoff of cell staining for defining HER2 positivity by ASCO/CAP recommendation in place from 2007 to 2013, all cases diagnosed in this time interval were reviewed by one expert pathologist to comply with the currently adopted 10% cutoff. In addition, 100 random matched cases underwent blinded revision by one expert pathologist, achieving an 85% agreement with the original report.

Estrogen receptor (ER) and progesterone receptor (PgR) expression were retrieved from the original pathology records. A 10% cutoff of ER/PgR expression was adopted for defining ER/PgR-positive cases. Cases were considered HR-positive in case of ER and/or PgR positivity, and negative in case of both ER and PgR negativity.

Residual proliferative cancer burden (RPCB) was evaluated in HR-positive/HER2-negative cohort by combining residual cancer burden (RCB) and post-treatment Ki67, as previously described^41,43^ and validated^44^. CPS-EG score was calculated based on clinical stage, pathological stage, grade, and ER status^45,46^.

### Statistical analysis

IBM SPSS Statistics (version 22.0) software (IBM Corp, Armonk, NY, USA) was used to carry out statistical analyses.

Patient demographics and clinical characteristics were reported by applying descriptive statistics. In particular, for continuous variables mean, median, range values, and quartiles were calculated. Distribution of continuous variables across subgroups was assessed by applying Student-*t* test, and The Mann–Whitney and Kolmogorov–Smirnov nonparametric tests. Chi-squared test (*χ*^2^) was applied to compare categorical variables across groups.

The evolution of HER2 expression from primary breast cancer to residual disease after neoadjuvant treatment was graphically reported by building Sankey diagrams. pCR was defined as the absence of invasive residual disease in both breast and lymph-nodes (ypT0/is, ypN0). DFS was defined as the time from diagnosis to recurrence or death from any cause. Survival curves were estimated by applying the Kaplan–Meier method and the log-rank test was used to test for differences between groups. The Cox-regression model was applied to calculate hazard ratios and 95% confidence intervals. All reported *p*-values are two-sided, and significance level was set at *p* < 0.05.

### Ethical statement

In the present study tumor samples were collected after approval by the Institutional Review Board (Istituto Oncologico Veneto I.O.V.–IRCCS, Padova) and in accordance with the Declaration of Helsinki. All patients provided written-informed consent prior to inclusion into the study.

### Reporting summary

Further information on research design is available in the Nature Research Reporting Summary linked to this article.

## Supplementary information

Supplementary information

Reporting Summary Checklist

## Data Availability

The datasets that support the findings of this study are not publicly available in order to protect patient privacy. The data will be available on reasonable request from the corresponding author: V.G., valentina.guarneri@unipd.it.
