# Supplementary material for: HER2-low-positive breast cancer: evolution from primary tumor to residual disease after neoadjuvant treatment
Source: NPJ Breast Cancer. 2022 May 20;8:66. doi: 10.1038/s41523-022-00434-w (PMC9122970; doi:10.1038/s41523-022-00434-w)
Supplement: Supplementary file 1 — Supplementary information [file 41523_2022_434_MOESM1_ESM.pdf]

Supplementary Figure 1

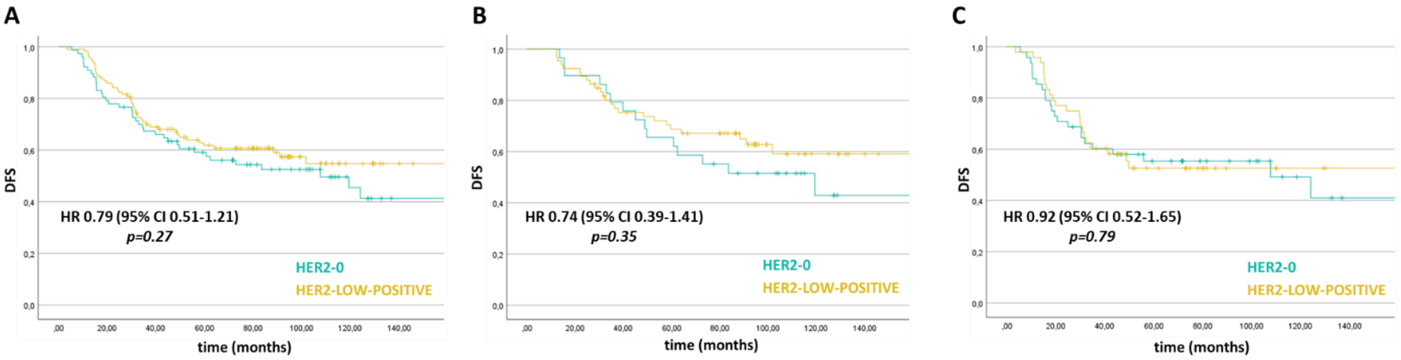

Kaplan Meier curves for disease-free survival (DFS) according to baseline HER2 expression. A, HER2-0 vs HER2-low-positive BC cohorts in the overall population; B HER2-0 vs HER2-low-positive BC cohorts in hormone receptor-positive/HER2-negative cohort; C HER2-0 vs HER2-low-positive BC cohorts in triple-negative cohort.

P values obtained by applying log-rank test and Cox regression model.

Supplementary Figure 2

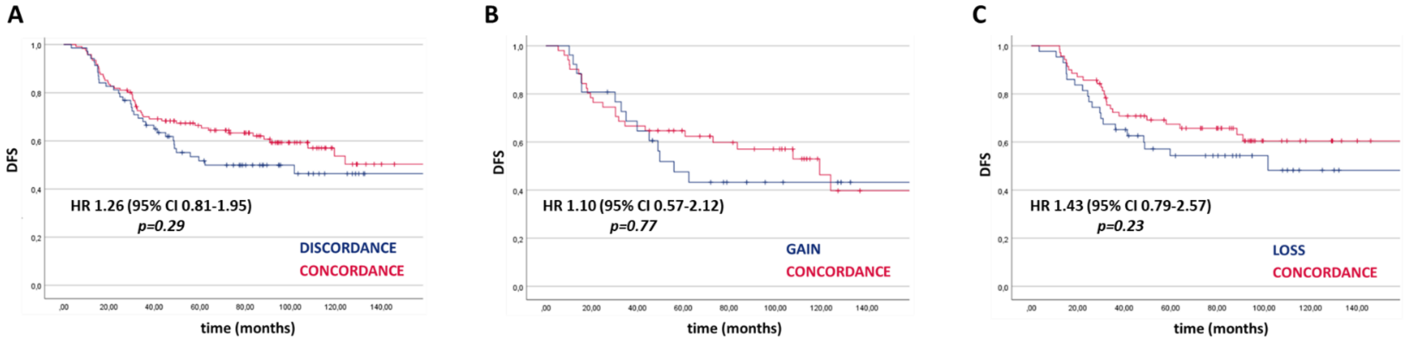

Kaplan Meier curves for disease-free-survival (DFS) according to HER2 expression change from baseline biopsy to residual disease after neoadjuvant treatment in HER2-negative cases. A, concordance vs discordance; B, concordant HER2-0 versus gain of HER2-low positive expression; C, concordant HER2-low positive versus loss of HER2-low positive expression.

P values obtained by applying log-rank test and Cox regression model.
