# Supplementary material for: HER2-low-positive breast cancer: evolution from primary tumor to residual disease after neoadjuvant treatment
Source: NPJ Breast Cancer. 2022 May 20;8:66. doi: 10.1038/s41523-022-00434-w (PMC9122970; doi:10.1038/s41523-022-00434-w)
Supplement: Supplementary file 2 — Reporting Summary Checklist [file 41523_2022_434_MOESM2_ESM.pdf]

nature portfolio

Corresponding author(s): VALENTINA GUARNERI

Last updated by author(s): Apr 5, 2022

# Reporting Summary

Nature Portfolio wishes to improve the reproducibility of the work that we publish. This form provides structure for consistency and transparency in reporting. For further information on Nature Portfolio policies, see our Editorial Policies and the Editorial Policy Checklist.

## Statistics

For all statistical analyses, confirm that the following items are present in the figure legend, table legend, main text, or Methods section.

| n/a | Confirmed | |
|---|---|---|
| ☐ | ☒ | The exact sample size (*n*) for each experimental group/condition, given as a discrete number and unit of measurement |
| ☐ | ☒ | A statement on whether measurements were taken from distinct samples or whether the same sample was measured repeatedly |
| ☐ | ☒ | The statistical test(s) used AND whether they are one- or two-sided<br>*Only common tests should be described solely by name; describe more complex techniques in the Methods section.* |
| ☐ | ☒ | A description of all covariates tested |
| ☐ | ☒ | A description of any assumptions or corrections, such as tests of normality and adjustment for multiple comparisons |
| ☐ | ☒ | A full description of the statistical parameters including central tendency (e.g. means) or other basic estimates (e.g. regression coefficient) AND variation (e.g. standard deviation) or associated estimates of uncertainty (e.g. confidence intervals) |
| ☐ | ☒ | For null hypothesis testing, the test statistic (e.g. *F*, *t*, *r*) with confidence intervals, effect sizes, degrees of freedom and *P* value noted<br>*Give P values as exact values whenever suitable.* |
| ☒ | ☐ | For Bayesian analysis, information on the choice of priors and Markov chain Monte Carlo settings |
| ☒ | ☐ | For hierarchical and complex designs, identification of the appropriate level for tests and full reporting of outcomes |
| ☒ | ☐ | Estimates of effect sizes (e.g. Cohen's *d*, Pearson's *r*), indicating how they were calculated |

*Our web collection on statistics for biologists contains articles on many of the points above.*

## Software and code

Policy information about availability of computer code

| | |
|---|---|
| Data collection | N/A |
| Data analysis | IBM SPSS Statistics (version 22.0), software (IBM Corp, Armonk, NY, USA) was used to carry out statistical analyses. |

For manuscripts utilizing custom algorithms or software that are central to the research but not yet described in published literature, software must be made available to editors and reviewers. We strongly encourage code deposition in a community repository (e.g. GitHub). See the Nature Portfolio guidelines for submitting code & software for further information.

## Data

Policy information about availability of data

All manuscripts must include a data availability statement. This statement should provide the following information, where applicable:
- Accession codes, unique identifiers, or web links for publicly available datasets
- A description of any restrictions on data availability
- For clinical datasets or third party data, please ensure that the statement adheres to our policy

The datasets that support the findings of this study are not publicly available in order to protect patient privacy. The data will be available on reasonable request from the corresponding author: VG, valentina.guarneri@unipd.it.

# Field-specific reporting

Please select the one below that is the best fit for your research. If you are not sure, read the appropriate sections before making your selection.

☒ Life sciences ☐ Behavioural & social sciences ☐ Ecological, evolutionary & environmental sciences

For a reference copy of the document with all sections, see nature.com/documents/nr-reporting-summary-flat.pdf

# Life sciences study design

All studies must disclose on these points even when the disclosure is negative.

| | |
|---|---|
| Sample size | 446 |
| Data exclusions | Patients with no HER2 expression data on baseline biopsy and, in case of no-pCR, surgigcal samples available |
| Replication | N/A |
| Randomization | N/A |
| Blinding | N/A |

# Reporting for specific materials, systems and methods

We require information from authors about some types of materials, experimental systems and methods used in many studies. Here, indicate whether each material, system or method listed is relevant to your study. If you are not sure if a list item applies to your research, read the appropriate section before selecting a response.

## Materials & experimental systems

| n/a | Involved in the study | |
|---|---|---|
| ☒ | ☐ | Antibodies |
| ☒ | ☐ | Eukaryotic cell lines |
| ☒ | ☐ | Palaeontology and archaeology |
| ☒ | ☐ | Animals and other organisms |
| ☐ | ☒ | Human research participants |
| ☐ | ☒ | Clinical data |
| ☒ | ☐ | Dual use research of concern |

## Methods

| n/a | Involved in the study | |
|---|---|---|
| ☒ | ☐ | ChIP-seq |
| ☒ | ☐ | Flow cytometry |
| ☒ | ☐ | MRI-based neuroimaging |

## Human research participants

Policy information about studies involving human research participants

| | |
|---|---|
| Population characteristics | 446 patients included<br>Age, median 50.2 (Q1-Q3: 42.7-60.2)<br>Hystology Ductal 397 89.0%; Lobular 28 6.3%; Other/NA 21 4.7%<br>Grading 1 4 0.9%; 2 89 20.0%; 3 316 70.9%; NA 37 8.2%<br>Clinical TNM I 21 4.7%; II 259 58.1%; III 159 35.7%; NA 7 1.5%<br>Primary BC phenotype HR+/HER2- 105 23.5%; TN 156 35.0%; HER2+ 185 41.5%<br>Neoadj. CT Anthra-Tax 354 79.4%; Tax 68 15.2%; Anthra 9 2.0%; Other/NA 15 3.4%<br>Neoadj. anti-HER2 Trastuzumab 160 35.9%;<br>Pathologic response pCR 155 34.8%; RD 291 65.2% |
| Recruitment | This represent a retrospective study including all consecutive BC patients undergoing neoadjuvant treatment. |
| Ethics oversight | Istituto Oncologico Veneto IOV - IRCCS, Padova, Italy. This study has been performed in accordance with the Declaration of Helsinki. All patients provided written-informed consent prior to inclusion into the study. |

Note that full information on the approval of the study protocol must also be provided in the manuscript.

## Clinical data

Policy information about clinical studies
All manuscripts should comply with the ICMJE guidelines for publication of clinical research and a completed CONSORT checklist must be included with all submissions.

| | |
|---|---|
| Clinical trial registration | N/A this is not a clinical trial |
| Study protocol | This is NOT a clinial trial. However, documents related to the study protocol submitted and approved by the abovementioned ethical committee are avaialble upon request |
| Data collection | This is NOT a clinical trial. Retrospective data collection in a dedicated database after data anonymization. |
| Outcomes | This is NOT a clinical trial. Retrispective study. The primary objective was to evaluate the evolution of HER2 expression from baseline biopsy to residual disease in patients undergoing neoadjuvant treatment. |
